# Inhibition of Rac1 reduces store overload‐induced calcium release and protects against ventricular arrhythmia

**DOI:** 10.1111/jcmm.12840

**Published:** 2016-05-25

**Authors:** Lili Zhang, Xiangru Lu, Le Gui, Yan Wu, Stephen M. Sims, Guoping Wang, Qingping Feng

**Affiliations:** ^1^ Institute of PathologyTongji HospitalTongji Medical CollegeHuazhong University of Science and TechnologyWuhanChina; ^2^ Department of Physiology and PharmacologyUniversity of Western OntarioLondonONCanada; ^3^ Nantong University Medical SchoolNantongChina; ^4^ Metabolic Syndrome Research CenterSecond Xiangya HospitalCentral South UniversityChangshaChina; ^5^ Lawson Health Research InstituteLondonONCanada

**Keywords:** Rac1, arrhythmia, store overload‐induced calcium release, ischaemia and reperfusion, reactive oxygen species

## Abstract

Rac1 is a small GTPase and plays key roles in multiple cellular processes including the production of reactive oxygen species (ROS). However, whether Rac1 activation during myocardial ischaemia and reperfusion (I/R) contributes to arrhythmogenesis is not fully understood. We aimed to study the effects of Rac1 inhibition on store overload‐induced Ca^2+^ release (SOICR) and ventricular arrhythmia during myocardial I/R. Adult Rac1^f/f^ and cardiac‐specific Rac1 knockdown (Rac1^ckd^) mice were subjected to myocardial I/R and their electrocardiograms (ECGs) were monitored for ventricular arrhythmia. Myocardial Rac1 activity was increased and ventricular arrhythmia was induced during I/R in Rac1^f/f^ mice. Remarkably, I/R‐induced ventricular arrhythmia was significantly decreased in Rac1^ckd^ compared to Rac1^f/f^ mice. Furthermore, treatment with Rac1 inhibitor NSC23766 decreased I/R‐induced ventricular arrhythmia. Ca^2+^ imaging analysis showed that in response to a 6 mM external Ca^2+^ concentration challenge, SOICR was induced with characteristic spontaneous intracellular Ca^2+^ waves in Rac1^f/f^ cardiomyocytes. Notably, SOICR was diminished by pharmacological and genetic inhibition of Rac1 in adult cardiomyocytes. Moreover, I/R‐induced ROS production and ryanodine receptor 2 (RyR2) oxidation were significantly inhibited in the myocardium of Rac1^ckd^ mice. We conclude that Rac1 activation induces ventricular arrhythmia during myocardial I/R. Inhibition of Rac1 suppresses SOICR and protects against ventricular arrhythmia. Blockade of Rac1 activation may represent a new paradigm for the treatment of cardiac arrhythmia in ischaemic heart disease.

## Introduction

Cardiac arrhythmia is a leading cause of death after myocardial infarction (MI) 1. Intracellular Ca^2+^ homoeostasis is critical to the heart in the maintenance of normal cardiac rhythms and contractility 2, 3, 4. Prolonged myocardial ischaemia during MI increases intracellular Ca^2+^, which is further exacerbated with reperfusion, leading to cardiomyocyte Ca^2+^ overload and cardiac arrhythmias 5, 6. Despite significant knowledge gained on cardiac Ca^2+^ cycling mechanisms, factors regulating cardiomyocyte Ca^2+^ and their roles in cardiac arrhythmia are still not fully understood.

Rac1 is a small GTPase protein and plays a key role in multiple cellular processes including cell migration, proliferation, survival and trafficking 7. Rac1 is also an essential regulatory subunit of NADPH oxidase for the generation of reactive oxygen species (ROS) 8, 9. In the heart, activation of Rac1 induces cardiac hypertrophy 10, 11, and genetic overexpression of Rac1 results in hypertension 12 and atrial fibrillation 13, 14. Rac1 activation also contributes to myocardial I/R injury in the diabetic hearts 15. However, the role of Rac1 in arrhythmogenesis is not fully understood.

Ryanodine receptor 2 (RyR2), an intracellular Ca^2+^ release channel in the sarcoplasmic reticulum (SR), is a key component in cardiac excitation‐contraction coupling. Under normal physiological conditions, a small Ca^2+^ influx through the L‐type Ca^2+^ channel upon membrane depolarization activates the RyR2 channel, resulting in a large Ca^2+^ release from the SR and subsequent muscle contraction 16. In the ischemic heart, phosphorylation and redox modifications of RyR2 increase RyR2 open probability causing SR Ca^2+^ leak 17, 18, 19. In addition, SR overload during myocardial I/R induces spontaneous Ca^2+^ release and leak mediated by RyR2, a term previously defined as store overload‐induced Ca^2+^ release (SOICR), leading to spontaneous Ca^2+^ waves in cardiomyocytes, which induce cardiac arrhythmias 20, 21, 22. Our recent studies show that activation of Rac1 increases intracellular Ca^2+^ in cardiomyocytes 23. However, how Rac1 disrupts Ca^2+^ homeostasis and promotes ventricular arrhythmia in the ischaemic heart remains elusive.

In this study, we hypothesized that inhibition of Rac1 protects against ventricular arrhythmia during myocardial I/R through decreases in ROS, RyR2 oxidation and SOICR. To test this hypothesis, cardiac‐specific Rac1 knockdown (Rac1^ckd^) mice were subjected to myocardial I/R. Rac1 activation, ROS production and oxidation of RyR2 were determined, SOICR in cardiomyocytes were analysed. Our data show for the first time that inhibition of Rac1 suppresses SOICR and protects against ventricular arrhythmia during myocardial I/R. Thus, Rac1 may represent a potential therapeutic target for the treatment of cardiac arrhythmia in ischaemic heart disease.

## Materials and methods

### Animals

Rac1^f/f^ mice with C57BL/6 background were obtained from Jackson Laboratory (Bar Habor, Maine, USA). In these mice, exon 1 of Rac1 gene is flanked by loxP sites. The generation of cardiac‐specific Rac1 knockdown mice (Rac1^ckd^, that is, Cre‐Tg;Rac1^f/f^) was carried out by breeding Rac1^f/f^ with cardiac‐specific α‐myosin heavy chain (α‐MHC) promoter controlled Cre recombinase overexpression transgenic mice (α‐MHC‐Cre), resulting in deletion of Rac1 gene in adult cardiomyocytes as previously described 23. The investigation conformed to the *Guide for the Care and Use of Laboratory Animals* published by the US National Institute of Health (8th Edition, 2011) and all experimental protocols were approved by Animal Use Subcommittee at the University of Western Ontario.

### Induction of myocardial I/R

Myocardial I/R were performed as previously described 24, 25. Briefly, Rac1^f/f^ and Rac1^ckd^ male mice (3–4 months old) were anaesthetized with ketamine (50 mg/kg, i.p.) and xylazine (12.5 mg/kg, i.p.). The adequacy of anaesthesia was monitored by the absence of withdrawal reflex to tail pinch. Subsequently mice were intubated and artificially ventilated with a respirator (SAK‐830, CWE, Ardmore, PA, USA). The left coronary artery was occluded by positioning a 8‐0 suture around it with a PE‐50 tubing. After 45 min., the PE tubing was removed to allow reperfusion for either 60 min. (Rac1 activation assay, ECG recording, infarct size and ROS generation measurement) or 15 min. (for free thiol content measurement). To study the effect of Rac1 inhibition, some Rac1^f/f^ and Rac1^ckd^ mice were treated with saline or a Rac1 inhibitor NSC23766 (2.5 mg/kg, IP) for 30 min. before I/R 26, 27.

### ECG monitoring

The ECG was monitored 5 min. before and throughout the entire I/R by limb lead I, with needle electrodes inserted subcutaneously in mice and recorded with PowerLab Chart 7.0 (AD Instrument, Colorado, Springs, CO, USA) as previously described 25. Premature ventricular contractions (PVCs) were defined as singlet or doublet premature QRS complexes in relation to the P wave. Ventricular tachycardia (VT) was defined as a run of three or more premature QRS complexes. The number of PVCs and incidence of VT were quantified. Additionally, PR interval, RR interval, QRS and QT durations were recorded.

### Infarct size measurement

After I/R, infarct size was determined as described previously 24, 28. In brief, after 45 min. ischaemia and 1 h reperfusion the left coronary artery was religated using the same suture. One per cent Evans blue dye (0.7 ml) was injected into the heart through the cannulated aorta to determine the non‐ischaemic (perfused, blue) and ischaemic (not perfused, not blue) areas of the heart. The heart was serially sectioned into four pieces and incubated with 1% triphenyltetrazolium chloride (TTC) for 10 min at room temperature. The viable, ischaemic tissue was stained into a red colour while infarct area was unstained as a white colour. Each heart section was pictured and weighed. The weight of non‐risk area (blue), infarct area (white) and area at risk (not blue) was calculated. Infarct size was measured as percentage of the weight of infarct area to the area at risk.

### Rac1 activity assay

Rac1 activity was measured by GTP‐bound Rac1 GTPase through specific protein interaction with the Pak1 protein‐binding domain (Active Rac1 Pull‐Down and Detection Kit; Thermo Scientific Pierce, Rockford, IL, USA) according to the manufacturer's instructions 29. Total homogenates of the heart tissue were centrifuged at 10,000 g for 10 min., and the supernatant was incubated with agarose beads coated with a GST‐Pak1 protein‐binding domain for 2 hrs at 4°C. The pulled down beads were resuspended in SDS sample buffer and boiled for 5 min. to elute Rac1‐GTP. Rac1‐GTP and total Rac1 protein levels were detected by western blot analysis.

### Western blot analysis

Protein lysates were subjected to separation on a 10% SDS‐PAGE gel, followed by electrotransfer to nitrocellulose membranes. Blots were probed with specific antibodies against Rac1 (Active Rac1 Pull‐Down and Detection Kit), RyR2 (MA3‐916, Affinity Bioreagents, Golden CO, USA), α‐actinin (A7811, Sigma, St. Louis, MO, USA) respectively. Signals were detected by the enhanced chemiluminescence detection method and quantified by densitometry using FluorChem 8000 software (Alpha Innotech, San Leandro, CA, USA).

### Isolation of adult cardiomyocytes

Adult cardiomyocytes were enzymatically isolated from the heart as described before 23. Briefly, mice were heparinized (5000 U/kg, i.p.) and killed by cervical dislocation under ketamine (50 mg/kg, i.p.) and xylazine (12.5 mg/kg, i.p.) anaesthesia. Hearts from Rac1^f/f^ and Rac1^ckd^ mice were isolated and perfused with digestion buffer containing 50 μg/ml of liberase TH (Roche) through a Langendorff system. After enzymatic digestion, healthy and rod‐shaped cardiomyocytes were incubated with buffer containing increasing concentrations of Ca^2+^ (12.5 μM–1 mM).

### Single cell Ca^2+^ imaging of SOICR

Ventricular cardiomyocytes isolated from Rac1^f/f^ and Rac1^ckd^ mice were loaded with 1 μM Fura‐2AM (Invitrogen, Waltham, MA, USA) for 30 min. at room temperature. To inhibit Rac1 activity, Rac1^f/f^ myocytes were perfused with a Rac1 inhibitor, NSC23766 (10 μM) 26, 27. SOICR was measured as previously described 21, 22. Briefly, cardiomyocytes were incubated in Krebs‐Ringer‐Hepes (KRH) buffer containing 1 mM CaCl_2_, 125 mM NaCl, 5 mM KCl, 6 mM glucose, 1.2 mM MgCl_2_ and 25 mM Hepes (pH7.4) and stimulated at 0.5 Hz for 20 sec. CaCl_2_ was added to the bath to final extracellular Ca^2+^ concentrations (1, 3 and 6 mM) followed by caffeine (10 mM) treatment to assess SR Ca^2+^ content.

### ROS production and RyR2 free thiol content

Changes in superoxide production from myocardium after I/R were detected with a fluorescent indicator dihydroethidium (DHE; Invitrogen) 30. In brief, fresh transverse croysections (8 μm) of the heart were placed on glass slides and incubated with DHE (2 μmol/l) for 30 min. at 37°C followed by washing with PBS for three times. Images were taken at a fixed exposure time under a fluorescence microscope (Observer D1, Zeiss, Germany) using an AxioCam high resolution camera. The fluorescence signal was measured with the AxioVision 4 program. RyR2 free thiols were determined with fluorescent probe for sulfhydryl (SH) groups of cysteines by monobromobimane (mBB) as described 18. Briefly, total homogenates of the risk area of the heart were prepared and heavy SR vesicles were isolated under non‐reducing conditions 18. Equal amount of protein samples were incubated with 500 μM mBB (Invitrogen) for 30 min. in the dark at room temperature. Samples were electrophoresed in 5% SDS‐PAGE gels and transilluminated with UV light. Monobromobimane fluorescence signal was normalized to total RyR2 measured by Coomassie blue staining of the gels run in parallel. The RyR2 band was confirmed by Western blotting using an anti‐RyR2 antibody.

### Reagents

Ketamine and xylazine were purchased from Bioniche Animal Health Canada Inc. (Belleville, ON, USA) and Bachem AG, Switzerland respectively. NSC23766 was from Calbiochem (San Diego, CA, USA). All other reagents if unspecified were obtained from Sigma, St. Louis, MO, USA.

### Statistical analysis

Data are presented as mean ± SEM. Results were analysed using one or two‐way anova followed by Newman–Keuls or Bonferroni *post‐hoc* test for multigroup comparisons. *P* < 0.05 was considered statistically significant.

## Results

### Rac1 activation during myocardial I/R

**Figure 1:**
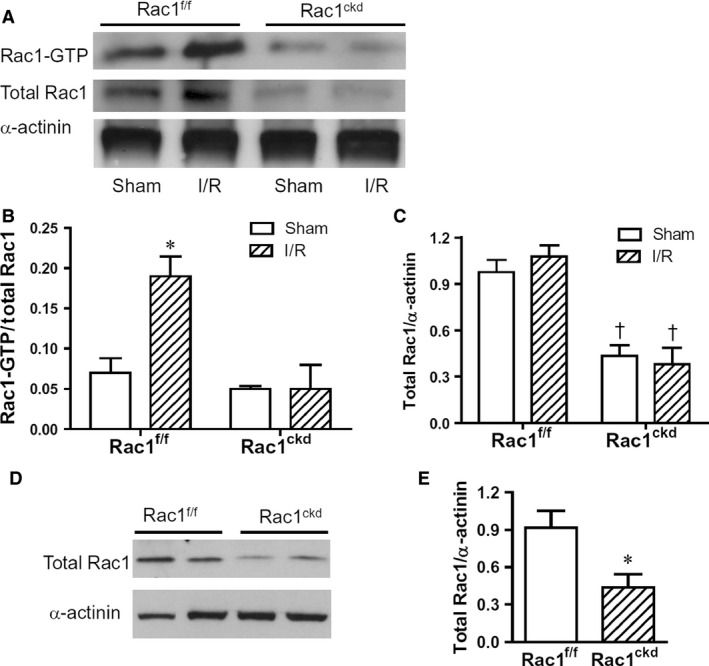
Myocardial Rac1 activity after I/R. (**A**) Representative Western blots of Rac1‐GTP with α‐actinin as loading control in Rac1^f/f^ and Rac1^ckd^ hearts after myocardial I/R. (**B**) Quantification of Rac1 activity using Rac1‐GTP to total Rac1 protein ratios. (**C**) Quantification of total Rac1 to α‐actinin protein ratios in the heart. (**D**) Representative Western blots of total Rac1 and α‐actinin proteins in isolated cardiomyocytes from adult Rac1^f/f^ and Rac1^ckd^ mice. (**E**) Quantification of total Rac1 to α‐actinin protein ratios in the isolated cardiomyocytes. Data are mean ± SEM from three to four mice per group. * *P* < 0.05 *versus* Rac1^f/f^ sham in B or Rac1^f/f^ in E, †*P* < 0.05 *versus* respective Rac1^f/f^.

Rac1^ckd^ mice were generated through breeding of alpha‐myosin heavy chain (MHC)‐Cre mice with Rac1^f/f^ mice and baseline cardiac function was not significantly different between adult Rac1^ckd^ and Rac1^f/f^ mice as we previously reported 23, 29. To assess myocardial Rac1 activity, Rac1^ckd^ and Rac1^f/f^ mice were subjected to 45 min. of myocardial ischaemia followed by 1 hr of reperfusion (I/R). Hearts were isolated, and Rac1‐GTP, total Rac1 and α‐actinin protein levels were determined using Western blot analysis. Myocardial Rac1 activity was induced after I/R in Rac1^f/f^ mice. The response was diminished in Rac1^ckd^ mice (Fig. 1A and B). Myocardial total Rac1 to α‐actinin protein ratios were decreased by 56% in Rac1^ckd^ compared to Rac1^f/f^ mice (Fig. 1C). In isolated cardiomyocytes, total Rac1 protein levels were reduced by 53% in Rac1^ckd^ compared to Rac1^f/f^ cells (Fig. 1D and E). These results show that a significant down‐regulation of total Rac1 protein and I/R‐induced Rac1 activity in Rac1^ckd^ mice.

### Knockdown of Rac1 inhibits ventricular arrhythmia during myocardial I/R

**Figure 2:**
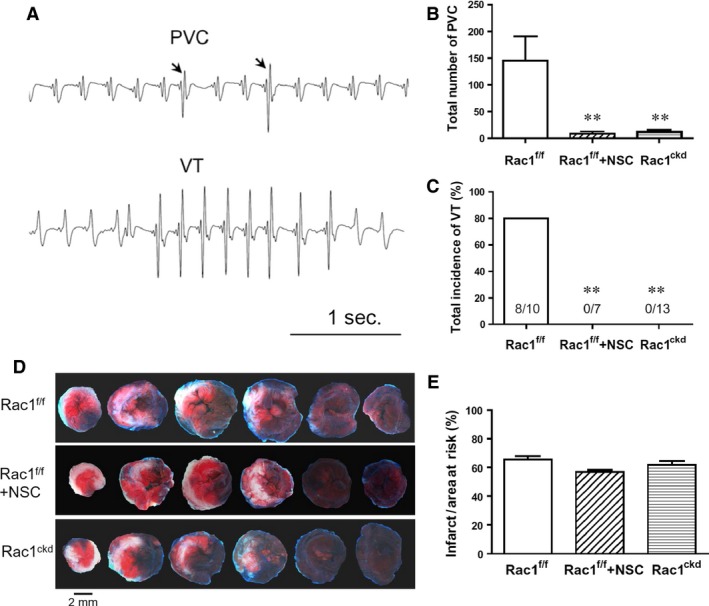
Inhibition of Rac1 decreases cardiac arrhythmia during 45 min. of ischaemia followed by 1 hr of reperfusion (I/R). (**A**) Representative ECG tracings showing premature ventricular complex (PVC) and ventricular tachycardia (VT). (**B**) Total number of PVC. (**C**) Total incidence of VT. (**D**) Representative sections of Evans Blue and triphenyltetrazolium chloride stained hearts showing area at risk (blue) and infarct area (white) respectively. (**E**) Quantification of infarct area to area at risk. Data are mean ± SEM. *n* = 7–13 mice per group in **B** and **C**,* n* = 6 mice per group in **E**. **P < 0.01 *versus* Rac1^f/f^.

To study the role of Rac1 in ventricular arrhythmia during I/R, Rac1^f/f^ and Rac1^ckd^ mice were subjected to 45 min. of myocardial ischaemia followed by 1 hr of reperfusion, and lead I ECG was monitored. Heart rate, PR interval, QRS duration and QTc interval were similar at baseline and during I/R between Rac1^f/f^, Rac1^f/f^ treated with NSC23766 and Rac1^ckd^ mice (Table 1). Notably, total number of ventricular premature beats (PVC), the incidence of VT and duration of VT were all significantly decreased in Rac1^f/f^ treated with NSC23766 and Rac1^ckd^ compared with Rac1^f/f^ mice (Fig. 2A and B, Table 2). These data show knockdown of Rac1 inhibits ventricular arrhythmias during myocardial I/R. However, infarct size determined at 1 hr after reperfusion was not significantly different among three groups (Fig. 2C and D), suggesting a similar level of ischaemic injury among the groups.

**Table 1:** Changes of heart rate, PR interval, QRS duration and QT interval following myocardial ischaemia and reperfusion

	Rac1^f/f^	Rac1^f/f^+NSC23766	Rac1^ckd^	*P* value
Mouse numbers	10	7	13	
Heart rate, bpm				
Basal	346 ± 20	332 ± 14	343 ± 24	0.917
Ischaemia	345 ± 20	352 ± 11	343 ± 18	0.946
Reperfusion	340 ± 18	359 ± 16	313 ± 15	0.173
PR interval, ms				
Basal	40.9 ± 0.8	41.4 ± 1.9	44.4 ± 1.0	0.798
Ischaemia	43.1 ± 3.6	45.1 ± 3.2	43.8 ± 4.2	0.239
Reperfusion	42.3 ± 1.5	51.2 ± 5.7	44.9 ± 1.7	0.958
QRS duration, ms				
Basal	21.3 ± 1.3	18.6 ± 2.1	21.6 ± 1.6	0.661
Ischaemia	22.6 ± 0.9	19.8 ± 1.4	21.3 ± 1.1	0.656
Reperfusion	21.4 ± 0.9	20.7 ± 1.1	22.1 ± 2.4	0.958
QTc interval, ms				
Basal	72.0 ± 1.9	64.9 ± 4.4	63.7 ± 3.3	0.264
Ischaemia	75.6 ± 1.6	68.5 ± 4.3	69.2 ± 3.3	0.250
Reperfusion	76.1 ± 2.0	74.2 ± 2.3	71.0 ± 2.2	0.230

Data are mean ± SEM. Mice were anaesthetized with ketamine and xylazine, and artificially ventilated with a respirator. Heart rate‐corrected QT interval (QTc) was calculated according to the formula QTc = QT/(RR/100)^1/2^. *P* values were analysed by one way anova. No significant difference was found in any of the parameters among three groups of mice.

**Table 2:** Inhibition of Rac1 reduces ventricular arrhythmia following myocardial ischaemia and reperfusion in mice

	Rac1^f/f^	Rac1^f/f^+NSC23766	Rac1^ckd^	*P* value
Mouse numbers	10	7	13	
Number of PVC				
Ischaemia	44.6 ± 14.3	2.0 ± 1.1**	6.3 ± 2.0**	0.0025
Reperfusion	100.2 ± 48.3	6.3 ± 3.3*	5.4 ± 2.9*	0.0351
Incidence of VT				
Ischaemia	3/10	0/7	0/13	0.0678
Reperfusion	5/10	0/7**	0/13**	0.0025
VT Duration, sec.				
Ischaemia	4.3 ± 0.3	0**	0**	0.0001
Reperfusion	3.9 ± 2.1	0**	0**	0.0001

Data are mean ± SEM. Mice were anaesthetized with ketamine and xylazine, and artificially ventilated with a respirator. *P* values on number of premature ventricular contraction (PVC) and ventricular tachycardia (VT) duration were analysed by one way anova followed by Newman–Keuls test. Incidence of VT was analysed by Chi‐square test. *P < 0.05, **P < 0.01 *versus* Rac1^f/f^ control mice.

### Inhibition of Rac1 decreases SOICR in cardiomyocytes

**Figure 3:**
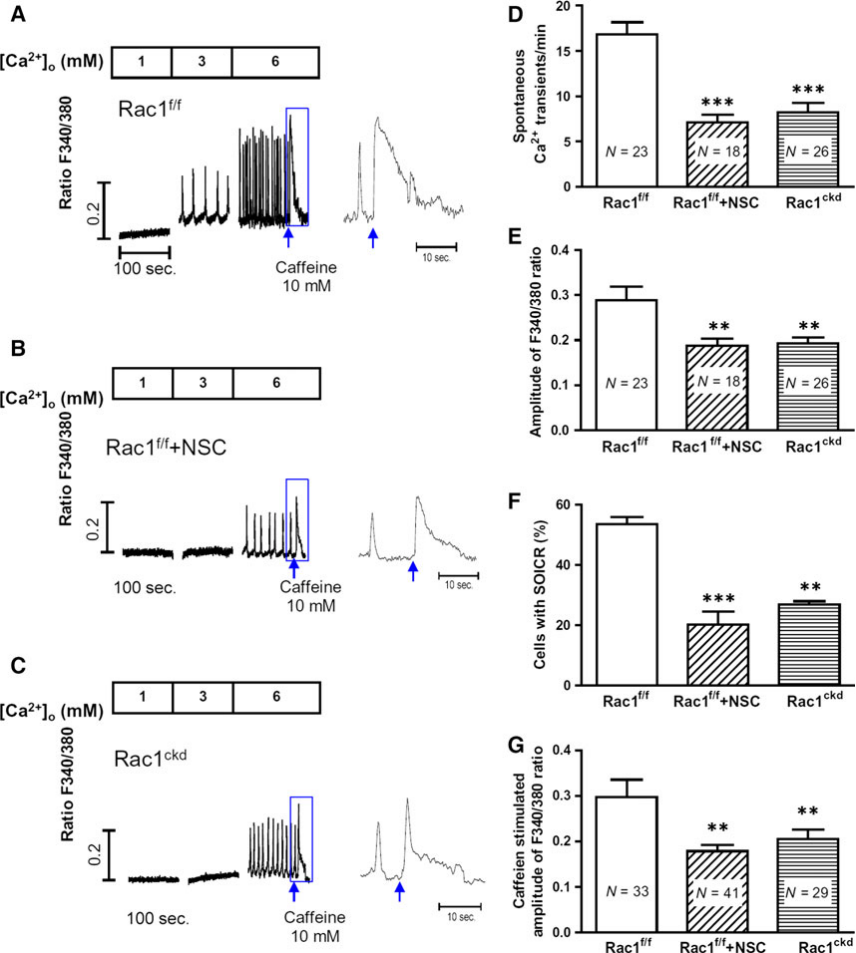
Effects of Rac1 inhibition decreases store overload‐induced Ca^2+^ release (SOICR) of adult ventricular myocytes. (**A**–**C**) Representative Fura‐2 ratios of Rac1^f/f^, NSC23766‐treated Rac1^f/f^ and Rac1^ckd^ myocytes in response to increasing extracellular Ca^2+^ concentrations (1, 3 and 6 mM) followed by caffeine (10 mM) stimulation (arrows). The boxed areas are enlarged. The threshold of SOICR was increased after Rac1 inhibition with NSC23766 and in Rac1^ckd^ myocytes. (**D**–**F**) Quantification of frequency, amplitude and occurrence of Ca^2+^ transients in Rac1^f/f^, NSC23766‐treated Rac1^f/f^ (Rac1^f/f^ +NSC) and Rac1^ckd^ cardiomyocytes in response to 6 mM extracellular Ca^2+^. (**G**) sarcoplasmic reticulum (SR) Ca^2+^ content assessed by caffeine stimulation. Data are mean ± SEM. Numbers in bars indicate myocyte numbers from three mice per group, **P < 0.01, ***P < 0.001 *versus* Rac1^f/f^.

Intracellular Ca^2+^ is increased in prolonged ischaemia. During reperfusion, SR Ca^2+^ ATPase activity is restored and increases Ca^2+^ uptake into the SR, leading to SR overload 4, 5. SOICR is a spontaneous SR Ca^2+^ release *via* RyR2 under conditions of SR Ca^2+^ overload, which is thought to be arrhythmogenic for interference of cardiac electrical activity 20, 21, 22. To study the role of Rac1 in Ca^2+^ release under SR overload, isolated Rac1^f/f^ and Rac1^ckd^ cardiomyocytes were loaded with fura‐2AM and Ca^2+^ concentration was monitored as the fluorescence ratio. Ca^2+^ store overload was induced in quiescent myocytes by elevating extracellular Ca^2+^ concentrations as previously described 31, and the occurrence of SOICR, that is, spontaneous intracellular Ca^2+^ waves, was monitored in single cells (Fig. 3A,B and C). The threshold extracellular Ca^2+^ concentration was ~3 mM for Rac1^f/f^ cardiomyocytes, whereas it was doubled at 6 mM for Rac1^ckd^ cardiomyocytes and for Rac1^f/f^ cardiomyocytes treated with a Rac1 inhibitor NSC23766. In response to 6 mM external Ca^2+^ concentration, the frequency and amplitude of SOICR were significantly decreased in Rac1^ckd^ and NSC23766‐treated Rac1^f/f^ cardiomyocytes (Fig. 3D and E). Ca^2+^ imaging confirmed that these events were calcium waves (see video S1 for normal uniform contractions in response to 0.5 Hz pacing in a Rac1^f/f^ myocyte and video S2 for spontaneous wave contractions of a Rac1^f/f^ myocyte in response to 6 mM extracellular Ca^2+^ concentration). Furthermore, at 6 mM external Ca^2+^ concentration, over 50% of Rac1^f/f^ cardiomyocytes exhibited SOICR while the occurrence of SOICR was seen in only 20–30% of Rac1^ckd^ and NSC23766‐treated Rac1^f/f^ cells (*P* < 0.01, Fig. 3F). SR Ca^2+^ content as assessed by caffeine (10 mM) stimulation at the end of 6 mM external Ca^2+^ treatment was significantly lower in Rac1^ckd^ and NSC23766‐treated Rac1^f/f^ cells (*P* < 0.01, Fig. 3G). These data show that Rac1 promotes SR Ca^2+^ release and induction of SOICR in response to Ca^2+^ overload.

**Figure 4:**
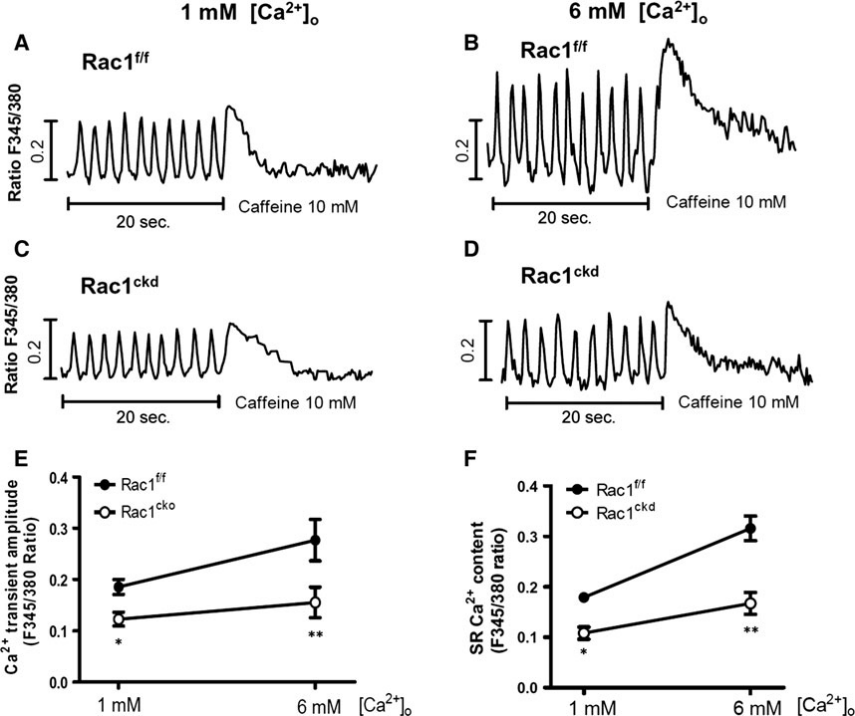
Rac1 inhibition reduces cardiomyocyte Ca^2+^ transients and sarcoplasmic reticulum (SR) Ca^2+^ load. (**A**–**D**) Representative Fura‐2 ratios in Rac1^f/f^ and Rac1^ckd^ myocytes, which were perfused at 1 and 6 mM extracellular Ca^2+^ concentrations and paced at 0.5 Hz for 20 sec. followed by treatment with caffeine. (**E**) Ca^2+^ transient amplitude in Rac1^f/f^ and Rac1^ckd^ myocytes during pacing. (**F**) SR Ca^2+^ load assessed by caffeine (10 mM) in Rac1^f/f^ and Rac1^ckd^ myocytes. Data are mean ± SEM of 10–16 myocytes from 3 mice per group, *P < 0.05, **P < 0.01 *versus* corresponding Rac1^f/f^.

To further assess changes in Ca^2+^ transients and SR Ca^2+^ content, myocytes were paced at 0.5 Hz followed by caffeine (10 mM) stimulation at 1 and 6 mM extracellular Ca^2+^ concentrations (Fig. 4A–D). Our data show that Ca^2+^ transients induced by pacing and SR Ca^2+^ content assessed by caffeine were significantly smaller in Rac1^ckd^ compared to Rac1^f/f^ myocytes (Fig. 4E and F).

### Inhibition of Rac1 reduces ROS generation and RyR2 oxidation during I/R

**Figure 5:**
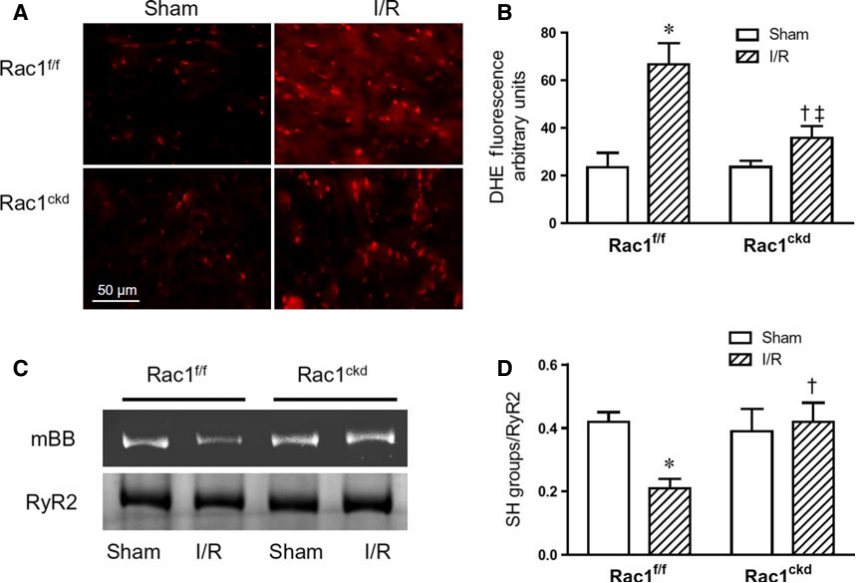
Rac1 inhibition decreases myocardial superoxide generation and oxidation of RyR2 after I/R. (**A**) Representative dihydroethidium (DHE) fluorescence staining (red) of heart sections after 45 min. of ischaemia followed by 1 hr of reperfusion (I/R). (**B**) Quantification of the red fluorescence intensity after I/R in Rac1^ckd^ and Rac1^f/f^ hearts. (**C**) Representative blots of RyR2 oxidation after 45 min. of ischaemia followed by 15 min. of reperfusion (I/R) using monobromobimane (mBB). (**D**) Quantification of free thiols of RyR2 in Rac1^f/f^ and Rac1^ckd^ hearts after I/R. Data are mean ± SEM. *N* = 3–5 mice per group, *P < 0.05 *versus* Rac1^f/f^ sham, †*P* < 0.05 *versus* Rac1^f/f^ I/R, ‡*P* < 0.05 *versus* Rac1^ckd^ sham.

Reactive oxygen species are generated in the heart during ischaemia and a significant burst of ROS formation occurs upon reperfusion 3, 4, 32. NADPH oxidases (Nox2 and Nox4) play a key role in ROS generation in the ischemic heart, and Rac1 is an important regulatory subunit of the Nox2 enzyme 33, 34. In the present study, superoxide generation during myocardial I/R was assessed using DHE staining on cryosections of the heart. The results show that myocardial I/R significantly increased ROS levels in Rac1^f/f^ hearts, which was reduced in Rac1^ckd^ hearts (Fig. 5A and B).

Since elevated oxidative stress induces RyR2 oxidation, which stimulates Ca^2+^ release, we propose that this could account for Ca^2+^ overload in cardiomyocytes 18, 35. Next we assessed RyR2 oxidation using monobromobimane (mBB), which labels free thiols of RyR2 18. Following myocardial I/R, mBB labelling was significantly decreased in Rac1^f/f^ hearts, and notably, the response was reversed in Rac1^ckd^ hearts (Fig. 5C and D). There were no significant changes in RyR2 band density (data not shown), suggesting equal RyR2 protein levels among all groups. The preservation of RyR2 free thiols is consistent with decreased ROS generation in Rac1^ckd^ hearts during I/R.

## Discussion

The major finding of this study is that inhibition of Rac1 protects against ventricular arrhythmia during myocardial I/R. We further demonstrated that genetic knockdown of Rac1 reduces SOICR in cardiomyocytes. Moreover, down‐regulation of Rac1 decreases ROS production and RyR2 oxidation during myocardial I/R. Our study suggests that Rac1 activation contributes to RyR2 oxidation, SOICR and ventricular arrhythmia during myocardial I/R. Thus, inhibition of Rac1 may have a therapeutic potential for clinical treatment of cardiac arrhythmia in ischaemic heart disease.

Intracellular Ca^2+^ homeostasis in cardiomyocytes is critical in maintaining normal electrical activities of the heart and Ca^2+^ overload is known to induce ventricular arrhythmia, cardiac hypertrophy and heart failure 5, 6, 32. Rac1 is a small GTPase involved in multiple physiological and cellular processes 7. Our recent studies show that activation of Rac1 increases intracellular Ca^2+^ in cardiomyocytes in response to cytokine stimulation 23. Myocardial I/R result in Ca^2+^ overload and cardiac arrhythmia 4, 5. However, the role of Rac1 in cardiomyocyte Ca^2+^ handling and ventricular arrhythmia during myocardial I/R remains elusive. We suggested that Rac1 is activated during myocardial I/R and inhibition of Rac1 protects against ventricular arrhythmia during I/R. To test this hypothesis, cardiac‐specific Rac1 knockdown mice were employed. We demonstrated that Rac1 is activated during myocardial I/R in Rac1^f/f^ mice and I/R‐induced Rac1 activity is decreased in Rac1^ckd^ mice. To monitor cardiac arrhythmia, ECG recordings were made during myocardial I/R. Our data show that the number of singlet and doublet ventricular premature beats and the incidence of ventricular tachycardia were significantly decreased by both pharmacological and genetic inhibition of Rac1. Importantly, infarct size following 45 min. of ischaemia and 1 hr of reperfusion was not significantly different among three groups of mice, indicating a similar level of cardiac injury. We did not assess infarct size beyond this period as most cardiac arrhythmia occurred within 40 min. after reperfusion. Thus, a beneficial effect of Rac1 inhibition on infarct size cannot be ruled out at a later time period. Collectively, these results suggest that activation of Rac1 in the heart contributes to ventricular arrhythmia during myocardial I/R. Previous studies showed that cardiac‐specific overexpression of constitutively active Rac1 induces atrial fibrillation in mice and higher Rac1 expression levels are associated with permanent atrial fibrillation in patients with atrial fibrosis 13, 14. Our study is the first demonstration of a definitive role of endogenous Rac1 activation in ventricular arrhythmogenesis during myocardial I/R. Studies have shown that NSC23766 inhibits M2 muscarinic receptors 36, and auto‐antibodies against M2 receptors, which enhance M2 receptor signalling, are associated with atrial fibrillation 37, 38. However, selective inhibition of M2 receptors has no significant effects on ischaemia‐induced ventricular arrhythmia 39. Therefore, the effects of NSC23766 on M2 receptor function are unlikely to contribute to the anti‐arrhythmic effects of NSC23766 observed during myocardial I/R in our study. Additionally, NSC23766 at 100 μM has been shown to have off‐target effects including inhibition of p21‐activated kinase (PAK) activity 40. In the present study, 2.5 mg/kg NSC23766 was used in mice, which is about 5 μM tissue concentration if it is evenly distributed in all tissues. This is 20 times lower than the concentration to achieve an off‐target effect. Thus, the anti‐arrhythmic effects of NSC23766 we observed are unlikely because of its off‐target effects.

To understand the mechanism by which Rac1 activation induces ventricular arrhythmia, SOICR, that is, spontaneous SR Ca^2+^ release *via* RyR2 under the conditions of SR Ca^2+^ overload, was investigated in this study. In response to increasing extracellular Ca^2+^ concentrations (3 and 6 mM), spontaneous intracellular Ca^2+^ oscillations were observed in both Rac1^f/f^ and Rac1^ckd^ cardiomyocytes, indicating induction of SOICR. However, the threshold extracellular Ca^2+^ to induce SOICR in Rac1^ckd^ cardiomyocytes was doubled. Furthermore, the frequency and amplitude of SOICR were significantly decreased in Rac1^ckd^ cardiomyocytes. Similar results were observed in Rac1^f/f^ cardiomyocytes treated with a pharmacological Rac1 inhibitor NSC23766. Our results demonstrated that Rac1 promotes the occurrence of SOICR in cardiomyocytes, which induces ventricular arrhythmia.

RyR2 is sensitive to redox modifications and oxidation of RyR2 by ROS generation induces SR Ca^2+^ leak in cardiomyocytes 18, 35. RyR2 is a tetrameric complex contains up to 89 cysteine residues per monomer, from which approximately 21 are redox sensitive 41. Rac1 is an important component of NADPH oxidase, which is a major ROS generating enzyme in the heart. In the present study, ROS production was significantly increased in Rac1^f/f^ hearts after I/R but was inhibited in Rac1^ckd^ mice. To assess thiol oxidation of RyR2, monobromobimane labelling assay was employed. Our data show that the amount of free thiols in RyR2 labelled by monobromobimane was significantly decreased after myocardial I/R in Rac1^f/f^ mice. Knockdown of Rac1 restored the amount of free thiols of RyR2. Our results suggest that Rac1 activation promotes ROS generation and RyR2 oxidation, leading to SOICR in cardiomyocytes during myocardial I/R. This study is limited to address the role of Rac1 in RyR2‐mediated cardiac Ca^2+^ handling. Further studies are required to examine whether Rac1 affects other key players including Na^+^/Ca^2+^ exchanger, SERCA and L‐type Ca^2+^ channels in cardiomyocytes during myocardial I/R.

Oberhofer *et al*. generated a mouse model that overexpresses a constitutively active mutant of Rac1, V12Rac1 in cardiomyocytes (RacET) 42. The RacET mice displayed cardiac dysfunction and dilated cardiomyopathy. Unexpectedly, a lower ROS production was found in cardiomyocytes. They further demonstrated that overexpression of V12Rac1 uncouples Rac1 activity from ROS production as a result of decreased p47^phox^ plasma membrane to cytosol ratio, which is critical to NADPH oxidase activity. The study showed that Rac1 overexpression results in a ROS‐independent Ca^2+^ dyshomeostasis and cardiac dysfunction 42. In our study, mice with cardiac‐specific knockdown of Rac1 show normal cardiac function 29. As a result of lower intracellular Ca^2+^ and SR Ca^2+^ content, the Rac1 knockdown cardiomyocytes are more resistant to Ca^2+^ overload during I/R injury. Data from our study are consistent with an important role of endogenous Rac1 in ROS‐dependent modulation of Ca^2+^ handling in cardiomyocytes.

In summary, this study showed that myocardial I/R activates Rac1 and induces ventricular arrhythmia. Inhibition of Rac1 decreases ROS production, RyR2 oxidation, and cardiac arrhythmia during I/R. Furthermore, SOICR, an important mechanism of arrhythmogenesis can be effectively inhibited by Rac1 inhibitor NSC23766 in cardiomyocytes. Collectively, our study demonstrated that inhibition of Rac1 activation protects against arrhythmogenesis during myocardial I/R, and may represent a new paradigm for the treatment of cardiac arrhythmia in ischemic heart disease.

## Funding

This study was supported by operating grants to Q.F. from the Canadian Institutes of Health Research (CIHR, MOP‐119600), to S.M.S. from CIHR (MOP‐64453) and to G.W. from National Natural Science Foundation of China (#81270176 and #81570254). L.Z. was a visiting PhD student from Tongji Medical College, Huazhong University of Science and Technology, Wuhan, China. Q.F. was a Career Investigator of the Heart Stroke Foundation of Ontario.

## Conflict of Interest

The authors declare that there are no conflicts of interest.

## Author contributions

S.M.S., G.W. and Q.F. contributed to conception and design of research; L.Z., X.L., L.G., and Y.W. performed experiments and analysed data. L.Z., L.G., S.M.S. and Q.F. interpreted results; L.Z. and L.G. prepared figures; L.Z. drafted manuscript; S.M.S., G.W. and Q.F. approved final version of manuscript; S.M.S. and Q.F. edited and revised manuscript.

## Supporting information


**Video S1.** Normal uniform contractions elicited by pacing isolated adult Rac1^f/f^ cardiomyocytes.
**Video S2.** Spontaneous waves in isolated adult Rac1^f/f^ cardiomyocytes in response to Ca2^+^ overload.
