# Supplementary material for: Inhibition of Rac1 reduces store overload‐induced calcium release and protects against ventricular arrhythmia
Source: J Cell Mol Med. 2016 May 25;20(8):1513–22. doi: 10.1111/jcmm.12840 (PMC4956946; doi:10.1111/jcmm.12840)
Supplement: Supplementary file 1 — Video S1. Normal uniform contractions elicited by pacing isolated adult Rac1f/f cardiomyocytes. Video S2. Spontaneous waves in isolated adult Rac1f/f cardiomyocytes in response to Ca2+ overload. [file JCMM-20-1513-s001.pdf]

Inhibition of Rac1 reduces store overload-induced calcium release and protects against ventricular arrhythmia

Lili Zhang, Xiangru Lu, Le Gui, Yan Wu, Stephen Sims, Guoping Wang, Qingping Feng

Supplemental videos

Videos of isolated adult cardiomyocytes were taken using an inverted Nikon Eclipse TE300 phase contrast microscope, and images were captured using Image Master 5 Software (Photon Technology International).

Supplemental Video 1. Normal uniform contractions elicited by pacing isolated adult $Rac1^{f/f}$ cardiomyocytes. Cardiomyocytes were incubated with Krebs-Ringer-Hepes (KRH) buffer containing 1 mM Ca^{2+}. Under control conditions myocytes are quiescent, and uniform contractions are evoked when cell is paced at 0.5 Hz. Video plays at twice actual speed. Width of frame is 270 μm.

Supplemental Video 2. Spontaneous waves in isolated adult $Rac1^{f/f}$ cardiomyocytes. Cardiomyocytes were incubated with Krebs-Ringer-Hepes (KRH) buffer containing 6 mM Ca^{2+} to induce Ca^{2+} overload and subsequent spontaneous Ca^{2+} release, which gives rise to Ca^{2+} waves and waves of contraction. Video plays at twice actual speed. Width of frame is 270 μm.
